# A Superhydrophobic Surface on a Superalloy Substrate with Properties of High Mechanical Strength and Self-Cleaning of Carbon Deposition

**DOI:** 10.3390/ma17020508

**Published:** 2024-01-20

**Authors:** Bingzhen Zhang, Yang Chen, Jinlong Song

**Affiliations:** 1 Key Laboratory for Precision and Non-Traditional Machining Technology of the Ministry of Education, Dalian University of Technology, Dalian 116024, China; zhangbingzhen@mail.dlut.edu.cn (B.Z.); c_yang93@126.com (Y.C.); 2 Key Laboratory for Micro/Nano Technology and System of Liaoning Province, Dalian University of Technology, Dalian 116024, China

**Keywords:** superhydrophobic, laser processing, mechanical strength, self-cleaning

## Abstract

Laser processing is an efficient method for fabricating a superhydrophobic surface and has attracted much attention due to its multifunctionality. However, excessive laser processing, such as laser beam overlap and multiple scans, generates both a thick, brittle recast layer and a thin material thickness, thereby greatly reducing the mechanical strength of the substrate. In addition, there is no report on fabricating a superhydrophobic surface on a superalloy substrate whose application includes a self-cleaning property. This work proposes the fabrication of a superhydrophobic surface on a superalloy substrate with high mechanical strength by optimizing the laser processing parameters including laser power, scanning speed, line spacing, and number of scans. We found that the microstructures required by superhydrophobicity could be constructed with a single laser scan. which could guarantee a minimal loss of the mechanical strength. The fabricated superhydrophobic surface on the superalloy substrate exhibited excellent self-cleaning of carbon deposition, showing good application potential in the aero engine field.

## 1. Introduction

In recent years, inspired by examples from nature such as the lotus leaf, roseleaf, and water skipper, researchers have paid increased attention to superhydrophobic surfaces because of their multifunctional properties such as self-cleaning [1,2,3,4], oil–water separation [5,6,7], droplet manipulation [8,9], and anti-icing [10,11]. Researchers have found that rough structures and low surface free energy are the two main factors for fabricating superhydrophobic surfaces and constitute an important strategy to obtaining superhydrophobicity by introducing a material containing liquid-repellent functional groups into artificial rough structures [12,13]. Water droplets show spherical as well as large contact angles (CAs) and low roll-off angles (RAs) on superhydrophobic surfaces, which follows the Cassie–Baxter wettability theory which holds that the contact between droplets and rough surfaces is composite and that air trapped in micro- and nanoscale structures prevents water droplets from collapsing and wetting surfaces. Generally, the more air is stored, the larger the droplet CAs on the surface. Therefore, constructing appropriate rough structures has a significant impact on the assurance of surface wettability.

To date, various methods, such as chemical etching, electrochemical deposition, and plasma etching, have been proposed to construct rough structures in different substrate materials including metal and polymer [14,15,16]. Rasitha et al. fabricated superhydrophobic titanium surfaces by rapid breakdown anodization and chemical modification with molten stearic acid. In their study, the sample surface showed TiO_2_ microclusters islands with hierarchical structures with nanopores and self-cleaning properties for graphite powder and myristic acid powder [17]. However, some disadvantages in terms of environmental pollution, sophisticated equipment, and difficulty in scaling up production still need to be addressed for further application. Laser processing is a method of constructing surface microstructures based on the thermal effect of laser beam and has significant prospects for the fabrication of superhydrophobic surfaces due to its environmentally friendly, high-energy utilization and less waste production, high efficiency, high precision, and repeatability [18,19]. According to the pulse duration, lasers can be classified into femtosecond, picosecond, and nanosecond lasers. Femtosecond lasers possess the shortest pulse duration, about 10^−15^ s, and there is nearly no thermal effect during material ablation. Pan et al. combined the methods of femtosecond laser ablation and chemical oxidation to design a triple-scale micronanostructured superhydrophobic surface on copper sheets, which exhibited excellent anti-icing and ice-phobic properties [20]. Compared with femtosecond lasers, picosecond lasers have a longer pulse duration (10^−12^ s), faster processing speed, and lower cost. Nguyen et al. used picosecond lasers to process enamel modified by fluorosilane agents and obtained superhydrophobic coatings with water CAs close to 180° [21]. Although nanosecond laser ablation generates a certain thermal effect due to the longer pulse duration (10^−9^ s), the features of cheap, high-efficiency and high-reliability keep nanosecond lasers competitive in large-scale industrial production. When the laser beam interacts with substrate, the energy is absorbed and converted into heat energy in a very short time, resulting in the ablation and removal of local material. Hence, nanosecond laser direct writing is usually employed to construct micronanostructures and fabricate superhydrophobic surfaces on aluminum [22,23], steel [24], stainless steel [25], titanium alloy [26], and so on. For example, Vanithakumari et al. fabricated wear-resistant superhydrophobic titanium surfaces using nanosecond laser patterning technology, which exhibited excellent antibacterial property against *Pseudomonas* sp. [27]. Boinovich et al. fabricated the superhydrophobic surface by 10 repetitions of laser texturing and one of fluorooxysilane chemisorption, improving the corrosion resistance of aluminum efficiently [28]. Song et al. fabricated microreentrant structures on copper plates successfully by combining 200 times of laser ablation with electrochemical deposition, and the prepared surface demonstrated CAs larger than 150° for both water and peanut oil [29]. Lee et al. fabricated a micropin array with a high aspect ratio on AISI 304 by repeating the laser beam ablation 2000 times [30]. However, excessive laser processing, namely repeated laser beam scanning, can cause serious defects such as recast layer and spatter, thereby reducing the mechanical strength of substrates and further limiting the application of superhydrophobic surfaces in structural components burdened with a heavy load. Consequently, it is of great importance to avoid excessive processing and obtain high strength structural components with superhydrophobicity by optimizing laser processing parameters, such as controlling the line spacing of laser beams or reducing the number of scans, which is also the focus of this work.

Superalloys have a wide range of applications in the aero engine field due to their low density and high strength [31]. However, carbon particles generated by fuel combustion often deposit on the surface of engines and become thicker with increasing service time, thus shortening the lifespan of engines and reducing the safety of aircrafts or automobiles [32,33,34]. Considering the excellent antifouling, antiadhesive, and self-cleaning performance of superhydrophobic surfaces (Table 1), if the superalloy surface is given extra superhydrophobicity, carbon deposits can be easily and efficiently rinsed off by water droplets during regular maintenance, and the engine performance can be restored in time. Generally, the contaminants used for self-cleaning tests are dust or powders of metals and metal oxides, which are deposited on superhydrophobic surfaces by gravity or smearing with low adhesion and are easily washed away by water. However, for carbon particles with smaller size, it is uncertain whether they can be cleaned off the surface. Moreover, there is no related literature on the application of laser processing technology in the fabrication of superhydrophobic superalloy surfaces.

In this paper, we propose fabricating a superhydrophobic superalloy surface using laser processing and the low-surface-energy modification method. To avoid excessive processing, the influence of laser processing parameters including power, scanning speed, and line spacing on the width and height of laser-affected regions and surface wettability was studied. By optimizing the processing parameters, superhydrophobicity could be obtained by single laser scanning, minimizing the effect of metal melting and sputtering on substrate strength. The mechanical strength of substrate under different processing times was also investigated with the tensile test. Finally, self-cleaning experiments of carbon deposition on the prepared surface were carried out, which included the self-cleaning of artificial carbon black and high-temperature carbon deposition.

## 2. Materials and Methods

### 2.1. Materials

Commercially available K424 nickel-based superalloy were purchased from Shenzhen Junhang Special Alloy Material Co. (Shenzhen, China). The chemical compositions of K424 superalloy used in the experiment are listed in Table 2. Ethanol was purchased from Tianjin Kermel Chemical Reagent Co. (Tianjin, China). Fluoroalkylsilane (FAS, tridecafluoroctyltriethoxysilane, CF_3_(CF_2_)_5_(CH_2_)_2_Si(OCH_3_)_3_) was purchased from Degussa Co. (Essen, Germany). All chemical reagents used in the experiment were analytically pure.

### 2.2. Fabrication of Superhydrophobic Superalloy Surfaces

**Figure 1:**
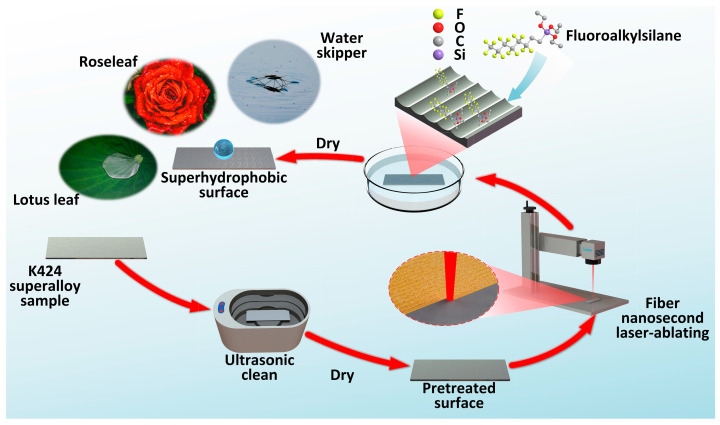
Fabrication processes of the superhydrophobic superalloy surface.

Figure 1 shows the fabrication processes of superhydrophobic superalloy surfaces. The superalloy sample was firstly cleaned with ethanol for 5 min using an ultrasonic cleaner (LT-05C, Hui Zhou Long Biao Electric Equipment Co., Ltd., Huizhou, China) to remove grease and other impurities on the surface. Then, the sample was placed horizontally on the workbench and ablated with a nanosecond laser mark system (wavelength of 1064 nm, repetition rate of 20 kHz, pulse duration of 100 ns, spot diameter of about 50 μm, focusing focal length of the optics and scanner head of 19.5 mm, M2 less than 1.3, maximum average power of 30 W, SK-CK30, 3k Laser Technology Co., Ltd., Shanghai, China) for the construction of rough structures. As the laser process parameters that produce superhydrophobic effects are discussed in the following section, the specific laser parameters are not described here. After being textured, the sample was cleaned again in ethanol for 5 min. Then, the cleaned sample was immersed into 1.0 wt.% FAS ethanol solution for 30 min and dried in an electrothermal constant-temperature oven (DHG-9023A, Jinghong Experimental Equipment Co., Ltd., Shanghai, China) at 80 °C for 10 min. Finally, the sample surface obtained superhydrophobicity. During the laser ablation process, a computer-controlled line-by-line scanning strategy was employed to focus and scan the high-energy laser beam onto the sample surfaces, utilizing an X-Y galvanometer. The sample size for exploring the wettability of superalloy surface was 30 mm × 50 mm × 5 mm. The processing length of the single-line texture was 10 mm, and the processing area size of the multiline texture was 10 mm × 10 mm. The properties of the superhydrophobic superalloy surface with laser processing parameters of 15 W-500 mm/s-50 μm were tested. The thickness of the superhydrophobic superalloy sample in the tensile test was 0.5 mm. The sample size for studying the stability and durability of the superalloy was 20 mm × 20 mm × 1 mm. The superhydrophobic superalloy surface was placed facedown onto a 2000# sandpaper under a weight of 50 g (the weight of the sample was 3.605 g, and the pressure was about 1.31 kPa), forcing the sample to rub on the sandpaper in the direction of the grooves (the velocity was about 0.1 m/s), and the CA and RA of the superhydrophobic superalloy surface were recorded for every 1 m of abrasion. The sample was not cleaned by water after each meter of abrasion, but the powder produced by the sandpaper was blown away with air. The sample size of the superhydrophobic superalloy surface used in the high temperature carbon deposition self-cleaning test was 20 mm × 20 mm × 0.1 mm.

### 2.3. Characterization

The micromorphology of the superhydrophobic surface and carbon deposition were observed with a scanning electron microscope (SEM, JSM-7900F, JEOL Ltd., Beijing, China). The width and height of the laser-affected region were observed with a 3D optical profiler (NewViewer 9000, ZYGO Corporation, Middlefield, CT, USA). For the surface microstructure obtained with the same laser processing parameters, at least five different areas were measured, and the standard deviation was used to describe the consistency of the microstructure morphology. The chemical elements of the superhydrophobic surface and the carbon deposition were characterized using a Fourier transform infrared spectrophotometer (FTIR, Nicolet 6700, Thermo Fisher Scientific Inc., Waltham, MA, USA), X-ray photoelectron spectroscopy (XPS, ESCALAB XI+, Thermo Fisher Scientific Inc., Waltham, MA, USA), and energy-dispersive X-ray spectroscopy (EDS, Ultim Max, Oxford Instruments plc, Oxford, UK). The surface wettability was characterized by static CAs and dynamic RAs. The CA and RA of 5 μL water droplets on the surperhydrophobic surface were observed with an optical CA meter (DSA100, KRÜSS Optronic GmbH, Hamburg, Germany), and the average value of five different positions was regarded as the final CA and RA. The mechanical strengths of the laser-processed specimens were characterized with an electronic universal testing machine (CTM2500, Xie Qiang Instrument Manufacturing Co. Ltd., Shanghai, China). All tests were carried out at room temperature.

## 3. Results

### 3.1. Microscopic Morphology of a Single Groove

**Figure 2:**
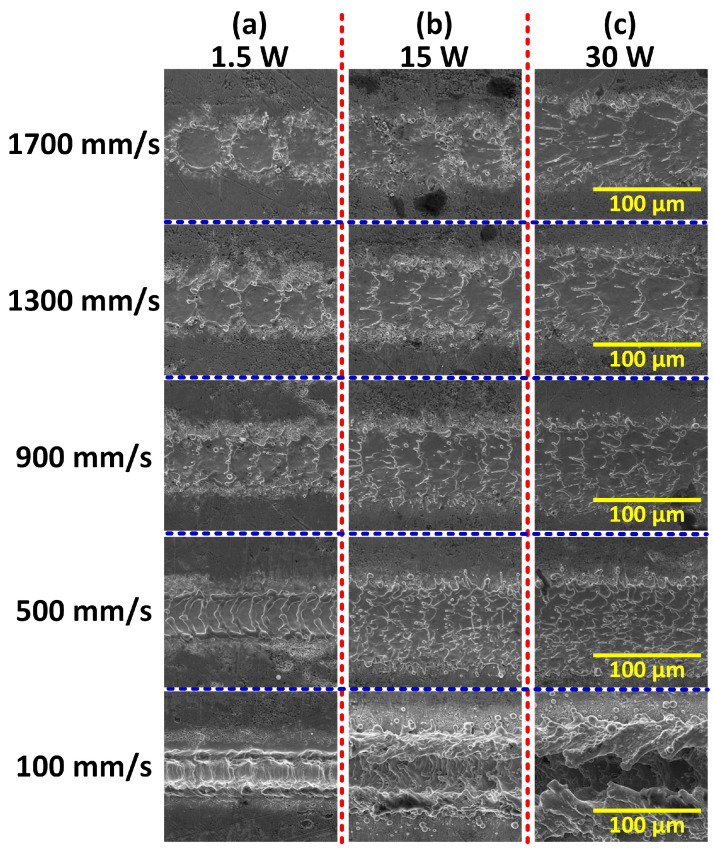
SEM images of the superalloy surface processed under different powers and scanning speeds.

Figure 2 shows the surface morphologies of the superalloy under different laser powers and scanning speeds. When the power was low and the scanning speed was high, circular 2D water-crown regions resulting from a single laser pulse were apparent, as shown in Figure 2a. In addition to the circular region ablated directly, the water-crown region also included a sputtering region formed by the solidification of the molten superalloy [42]. With the decrease in scanning speed, the water-crown regions overlapped each other, and the whole processing region converted to a groove shape. Moreover, the size of the single-pulse processing region increased with the power, resulting in larger overlapping area and wider processing region under the same scanning speed, as shown in Figure 2c.

**Figure 3:**
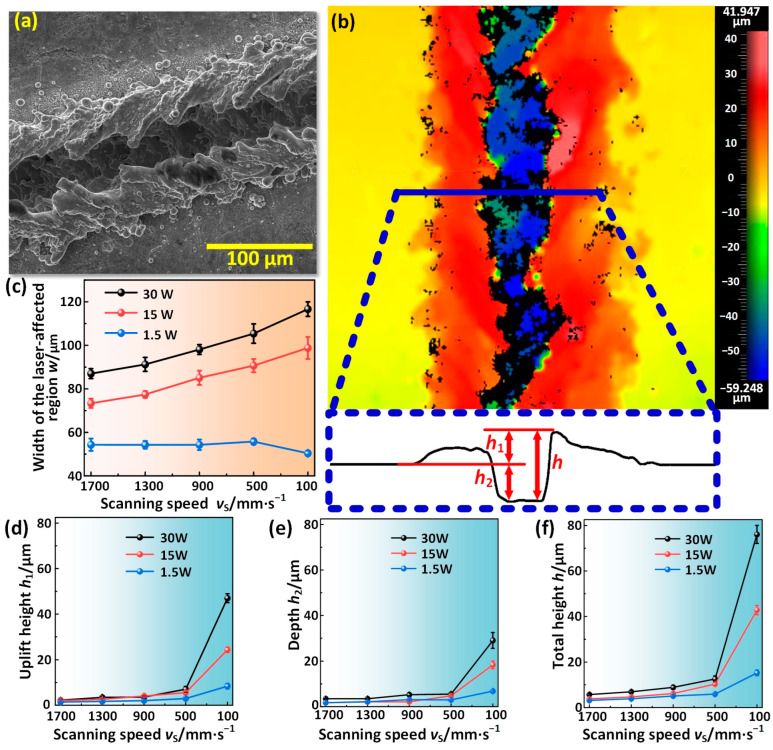
Variations of the microscopic geometric features of the laser-affected region with power and scanning speed. (**a**,**b**) SEM image and 3D profile of a single groove. (**c**–**f**) Variations of the width *w*, uplift height *h*_1_, depth *h*_2_, and total height *h* of the laser-affected region with power and scanning speed, respectively.

Figure 3 shows the variations of the microscopic geometric features of the laser-affected region with power and scanning speed. The laser-affected region comprised ablated and sputtered regions, and its representative cross-section morphology including protrusion and depression is shown in Figure 3b. Figure 3c shows the variation of the laser-affected region width *w* with power and scanning speed. It can be seen that the *w* increased with the decrease in scanning speed when the power was 15 W and 30 W. For example, with the scanning speed decreased from 1700 mm/s to 100 mm/s, the *w* increased from 73.4 ± 2.1 μm to 98.4 ± 5.0 μm at 15 W power and increased from 87.0 ± 2.3 μm to 116.7 ± 3.4 μm at 30 W power. The reason for this was that the lower the scanning speed was, the longer the laser irradiation time was, resulting in more energy absorption and more serious ablation and sputtering. However, the *w* hardly changed when the power was 1.5 W, only decreasing from 54.3 ± 2.8 μm at a scanning speed of 1700 mm/s to 50.4 ± 1.2 μm at a scanning speed of 100 mm/s. The power was too low to form a large amount of sputtering, and the *w* was only slightly larger than spot diameter *d*. Furthermore, it can be seen that the *w* always increased with power at any scanning speed.

Figure 3d–f show the variations of the height and depth of the laser-affected region with power and scanning speed. To express this more intuitively, we defined the height of the protrusion and the depth of the depression as *h*_1_ and *h*_2_, respectively, and the height from the highest point to the lowest point as *h*, namely *h* = *h*_1_ + *h*_2_ (Figure 3b). The protrusion was primarily formed by the sputtering accumulation of molten superalloy, and the depression was the laser-ablated region. *h*_1_, *h*_2_, and h increased with the decrease in scanning speed. When the scanning speed was 1700 mm/s, *h*_1_, *h*_2_, and *h* were (3.5 ± 0.2 μm, 2.2 ± 0.3 μm, 5.7 ± 0.5 μm), (1.7 ± 0.1 μm, 2.1 ± 0.1 μm, 3.8 ± 0.2 μm), and (1.7 ± 0.2 μm, 1.4 ± 0.1 μm, 3.1 ± 0.3 μm) under the power of 30 W, 15 W, and 1.5 W, respectively. Nevertheless, when the scanning speed was 100 mm/s, *h*_1_, *h*_2_, and *h* increased to (29.0 ± 3.4 μm, 47.0 ± 2.0 μm, 76.1 ± 5.4 μm), (18.4 ± 1.7 μm, 22.4 ± 1.2 μm, 42.8 ± 2.8 μm). and (6.8 ± 0.4 μm, 8.4 ± 1.1 μm, 15.2 ± 1.6 μm) under the power of 30 W, 15 W, and 1.5 W, respectively. Additionally, it can be seen from Figure 3d–f that higher power would contribute to larger values of *h*_1_, *h*_2_, and *h*. The main reason for this phenomenon was that the higher power or the lower scanning speed was, the more laser energy was absorbed by a specific region on the superalloy surface, resulting in the more serious ablation and sputtering.

### 3.2. Wettability of the Surface with Groove Arrays

**Figure 4:**
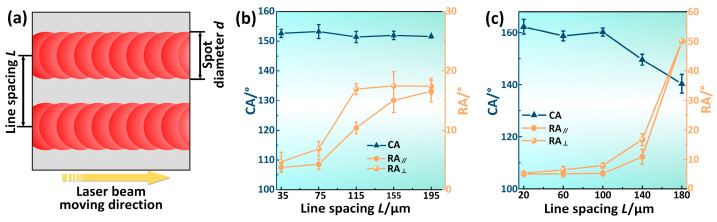
The effect of line spacing on the wettability of the laser-processed surfaces. (**a**) Schematic diagram of the *w*, spot diameter *d*, and line spacing *L*. (**b**) Variations of the CA and RA with a line spacing under 30 W-100 mm/s for the laser processing parameters. (**c**) Variations of the CA and RA with a line spacing under 30 W-500 mm/s for the laser processing parameters.

A single laser scanning accounted for the formation of micro/nanostructures required for superhydrophobicity. However, to obtain superhydrophobicity, the processing region needed to be spread over the entire surface through line-by-line scanning. If the line spacing between the two adjacent laser-affected regions was no larger than the *w*, the micro/nanostructures could be constructed successfully on the entire surface after laser scanning, which was expected to obtain superhydrophobicity, as shown in Figure 4a. In addition, to avoid repeated processing, line spacing was required to be larger than *d*, i.e., 50 μm. An experiment was conducted to verify the above-mentioned hypothesis. For comparison, the CA of the surface without laser processing is about 98°, and the water droplets cannot roll. Figure 4b shows the variations of the CA and RA of water droplets on the fluorinated surface over a line spacing under 30 W-100 mm/s for the laser processing parameters. For a line spacing ranging from 75 μm to 195 μm, CAs remained stable, varying from 151.4 ± 1.9° to 153.2 ± 2.4°, but the RAs varied considerably. The RAs parallel to the groove direction RA_‖_ and perpendicular to the groove direction RA_⊥_ were both less than 10° when the line spacing was 75 μm; RA_‖_ was 10.4 ± 0.9° while RA_⊥_ was 16.9 ± 0.9° when the line spacing was 115 μm; RA_‖_ and RA_⊥_ were both larger than 15° when the line spacing was 155 μm. For the laser processing parameters of 30 W-100 mm/s, the *w* was nearly 108 μm. When the line spacing was less than 108 μm, the adjacent processing regions overlapped, leading to complete coverage of the micro/nanostructures on the entire surface; however, when the line spacing was larger than 108 μm, some areas were not covered by micro/nanostructures and thus did not show superhydrophobicity. A similar phenomenon could also be found under the laser processing parameters of 30 W-500 mm/s. When the line spacing was less than the *w* of 105 μm, the surface showed excellent superhydrophobicity with a high water CA and a low RA; however, the superhydrophobicity gradually disappeared with the increase in the line spacing, as shown in Figure 4c. In summary, to ensure the superhydrophobicity of the surface and avoid repeated processing, the line spacing should be larger than the *d* and less than the *w*.

In addition to the laser processing parameters of 30 W-100 mm/s and 30 W-500 mm/s, other parameters were also assessed to verify the above-mentioned conclusion. Table 3. shows the CAs and RAs of water droplets under different laser processing parameters, where the line spacing was less than the *w*. The results showed that none of the surfaces could obtain superhydrophobicity except for the surface with *h* larger than 10 μm, which is consistent with the Cassie–Baxter wettability theory which holds that a certain amount of internal space is required to lock the air cushion in supporting the water droplet. Therefore, appropriate conditions, such as a line spacing less than the *w* and *h* larger than 10 μm, were beneficial for the formation of superhydrophobicity. In our study, the sufficient processing parameters were as follows: 30 W-100 mm/s, 30 W-500 mm/s, 15 W-100 mm/s, 15 W-500 mm/s, and 1.5 W-100 mm/s.

**Figure 5:**
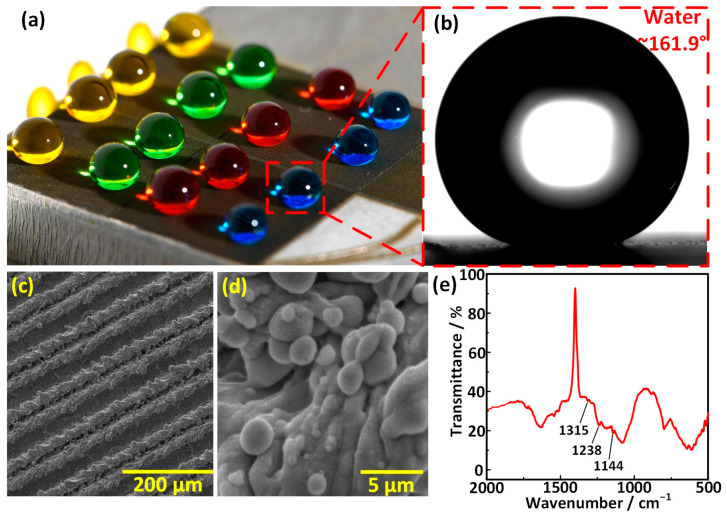
Superhydrophobic superalloy surface. (**a**) Digital image of the water droplets on the superhydrophobic superalloy surface. (**b**) The image of a water droplet on the superhydrophobic superalloy surface. (**c**,**d**) SEM images of the superhydrophobic superalloy surface with different magnifications. (**e**) FTIR spectrum of the surperhydrophobic superalloy surface.

Figure 5a shows the digital image of water droplets on the superhydrophobic superalloy surface processed under the laser processing parameters of 15 W-100 mm/s-100 μm. The water droplet exhibited a typical spherical shape with a CA of 161.9°, as shown in Figure 5b. SEM images with different magnifications showed that the processed surface was covered by groove arrays and that the bottom, top, and sides of the entirety of the grooves were covered with multiple-scale micro/nanostructures whose sizes ranged from 300 nm to 5 μm, as shown in Figure 5c,d. It could be seen from the FTIR spectrum that C-F bond stretching vibration peaks of -CF_2_- functional group and -CF_3_ functional group in fluoroalkylsilane were detected at 1315 cm^−1^, 1238 cm^−1^, and 1144 cm^−1^, indicating the fluoroalkylsilane film with low surface energy covered the rough micronanostructures successfully (Figure 5e) [43,44]. The combination of rough trans-scale micronanostructures and low surface energy contributed to endowing the superalloy surface with superhydrophobicity.

### 3.3. Effect of Laser Processing on Surface Mechanical Strength

**Figure 6:**
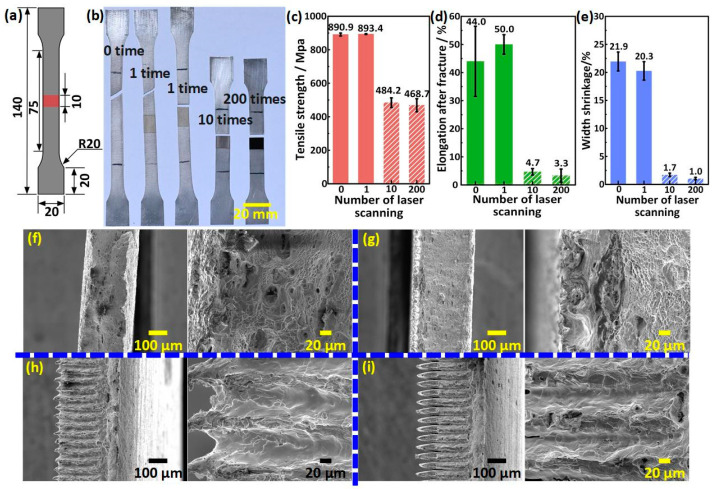
Effect of the number of laser scanning on the mechanical strength of superhydrophobic superalloy. (**a**) Schematic illustration of the shape and size of the specimen (unit: mm). (**b**) Photo of the specimens processed with a different number of laser scans after the tensile test. (**c**–**e**) Variations of tensile strength, elongation, and width shrinkage of the specimens with the number of laser scans, respectively. (**f**–**i**) The micromorphology of the fracture processed under a different number of laser scans: (**f**) 0 times, (**g**) 1 time, (**h**) 10 times, and (**i**) 200 times.

Superhydrophobicity can be obtained by laser processing technology with single or multiple scans; however, in practical application, the mechanical strength of the superalloy might be affected by the number of laser scans. Therefore, the effect of the number of laser scans on the mechanical strength of the superalloy was studied with the tensile test. In addition to scanning the surface once, other amounts of scans listed in the existing literature were chosen for comparison, such as 10 and 200 [28,29]. The schematic illustration of stretched specimens with a gauge length of 50 mm, an original width of 12.5 mm, and a laser processing region length of 10 mm is shown in Figure 6a. Figure 6b shows the photo of the stretched specimens with different numbers of scans after the tensile test, and it can be seen that the fracture of the ordinary specimen without laser scanning was serrated while the fracture of the specimens under 10 and 200 laser scans was planar. For the specimens scanned once, some fractures were serrated while others were planar. In addition, the planar fractures were all located at the boundary between the laser scanning area and the unprocessed area, which conformed to the characteristics of brittle fracture. Furthermore, the tensile strength, elongation, and width shrinkage of the specimens scanned once were close to those of the specimens that were not scanned. The tensile strength, elongation, and width shrinkage of the specimens scanned 10 times and 200 times only reached 54.2% and 52.5%, 9.4% and 6.6%, and 8.4% and 4.9%, respectively, of those of the specimens scanned once, as shown in Figure 6c–e. Figure 6f–i show the micro-morphology of the fracture processed under different numbers of laser scans. The fracture surfaces of the specimens not scanned and those scanned once were covered with dimples, which was consistent with plastic fracture; meanwhile, the fracture surfaces of the specimens scanned 10 times and 200 times were covered with grooves, and the inner surfaces of the grooves were smooth, which was more consistent with brittle fracture. In summary, excessive scanning could decrease the mechanical strength significantly by thinning the material thickness and thickening the melt-solidified layer, and a superhydrophobic surface scanned once could obtain the best mechanical strength.

### 3.4. Stability and Durability of the Superhydrophobic Superalloy Surface

**Figure 7:**
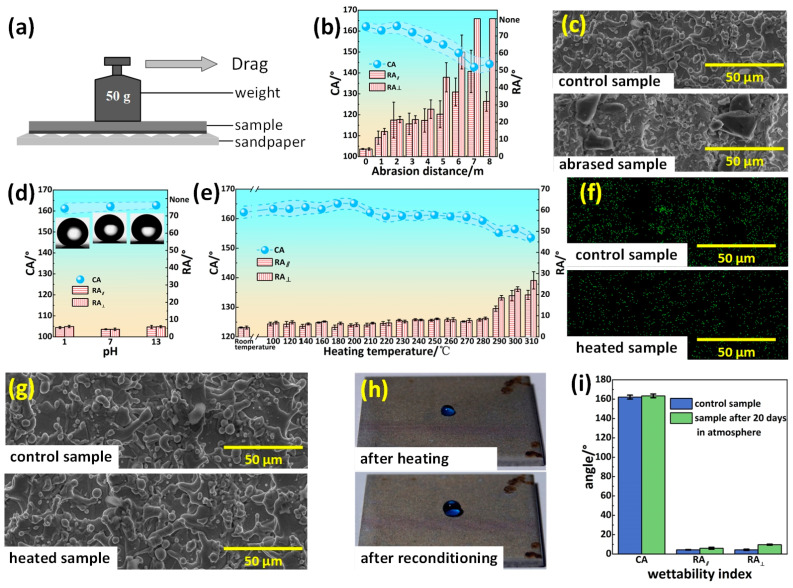
Stability and durability of superhydrophobic superalloy sample. (**a**) Schematic diagram of the sandpaper abrasion test. (**b**) Variation of the CA and RA with abrasion distance. (**c**) SEM images of the samples before and after abrasion test. (**d**) Variation of the CA and RA with temperature. (**e**) EDS element mappings of fluorine of the sample before and after heating treatment. (**f**) SEM images of the sample before and after heating treatment. (**g**) Photographs of the water droplets on the surface of the sample before and after reconditioning. (**h**) Variation of the CA and RA of the sample with time. (**i**) The CA and RA of the sample before and after being exposed to the atmosphere for 20 days.

Although the superhydrophobic superalloy surface showed excellent mechanical strength, the robustness and stability of the surface remained to be explored for practical applications. To examine the robustness and stability of the superhydrophobic superalloy surface, sandpaper abrasion and thermal stability tests were conducted. The sandpaper abrasion test device is shown in the Figure 7a. The result of the sandpaper abrasion experiment depicted in Figure 7b showed that the superhydrophobic superalloy surface could maintain excellent superhydrophobicity with a CA above 150° and an RA below 25° after undergoing 4 m of abrasion. This indicates that the superhydrophobic superalloy surface exhibits strong resistance to mechanical abrasion. To further study the change of surface after sandpaper abrasion, SEM images helped to illustrate the details of the microstructure. After 4 m sandpaper abrasion, the microscale structures on the sample surface were only slightly damaged, as shown in Figure 7c.

To evaluate the chemical stability of the superhydrophobic superalloy surface, we dropped 5 μL of HCl aqueous solution (pH = 1), water (pH = 7), and NaOH aqueous solution (pH = 13) on the modified sample surface. All three droplets exhibited significantly high CAs on the superhydrophobic surface, with an angle of 161.18 ± 2.56° for HCl aqueous solution, 162.16 ± 2.06° for water, and 162.74 ± 1.55° for NaOH aqueous solution, as shown in Figure 7d. Furthermore, these droplets displayed low RAs and could effortlessly roll off the superhydrophobic superalloy surface, indicating its excellent resistance against strong acidic and alkaline liquids.

The heat treatment test was also performed to determine the thermostability of the superhydrophobic superalloy surface [45]. The superhydrophobic superalloy surface was put on a heating platform with different operating temperatures for 30 min. Figure 7e shows the change of surface superhydrophobicity with temperature. In the temperature range of 20–280 °C, the CA and RA of the superhydrophobic superalloy surface had almost no change, with the CA being larger than 160° and the RA being smaller than 10°. When the temperature further increased, the droplet CA on the surface slowly decreased and the RA gradually increased. The EDS mapping image shown in Figure 7f shows that the content of F element on the ablated surface was lower than that on the original surface, indicating that the fluorosilane layer on the surface was partially destroyed. Since there was no damage to the surface of the micro/nanostructure, whose SEM image is shown in Figure 7g, the surface recovered superhydrophobicity after being immersed in fluorosilane ethanol solution (Figure 7h).

In order to test the durability of the superhydrophobic superalloy surface, the samples were exposed to the atmosphere for 20 days, and the changes in the CA and RA were recorded. The comparison of the CA values and RA values of the samples before and after the exposure is shown in Figure 7i. The CA changed to 163.35 ± 1.93°, and the RA_‖_ and RA_⊥_ increased to 5.67 ± 0.76° and 9.67 ± 0.58°, respectively. The CA and RA of the samples before and after 20 days of exposure to the atmosphere changed very little, so the hydrophobicity of the superhydrophobic superalloy surface could be maintained for an extended period of time. In conclusion, from the aspect of practical application, the laser processing of superhydrophobic superalloy surface has broad application prospects.

### 3.5. Self-Cleaning of Carbon Deposition

Apart from the excellent properties mentioned above, a self-cleaning property would be an important criterion for further evaluating the application potential of the superhydrophobic surface. Thus, the superhydrophobic superalloy surfaces processed under the laser processing parameters of 15 W-500 mm/s-50 μm were used to carry out self-cleaning experiments, including a self-cleaning experiment of artificial carbon black and a self-cleaning experiment of high-temperature carbon deposition.

#### 3.5.1. Self-Cleaning of Artificial Carbon Black

**Figure 8:**
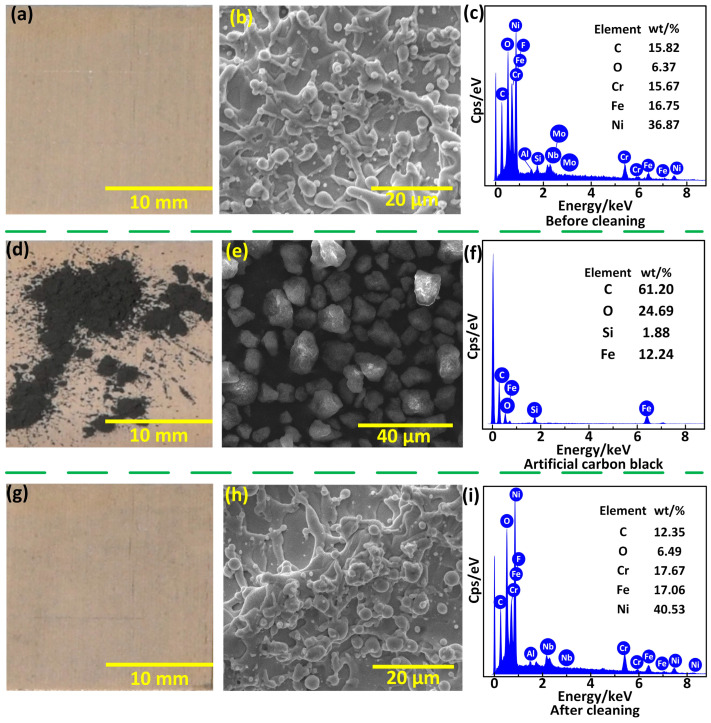
Self-cleaning of artificial carbon black. (**a**,**b**) Macro- and micromorphology of the superhydrophobic superalloy surface. (**c**) EDS of the superhydrophobic superalloy surface. (**d**,**e**) Macro- and micromorphology of the superhydrophobic superalloy surface with carbon black deposit. (**f**) EDS of carbon black. (**g**,**h**) Macro- and micromorphology of the superhydrophobic superalloy surface after the self-cleaning experiment. (**i**) EDS of the superhydrophobic superalloy surface after the self-cleaning experiment.

The superhydrophobic surface fabricated under the laser processing parameters of 15 W-500 mm/s-50 μm presented as apricot-colored macroscopically, as shown in Figure 8a. Figure 8b shows the SEM image of the surface, and the surface was covered with micro/nanostructures formed by the deposition of a molten superalloy. In addition, the EDS spectrum shown in Figure 8c indicated that the main elements of the superhydrophobic surface were Ni, Cr, Fe, and O, among others. A self-cleaning experiment was carried out by depositing carbon black on the superhydrophobic surface artificially (Figure 8d) and then dropping water droplets with a volume of 53 μL (the flow rate was ~12 mL/min) on the surface continuously to rinse the carbon black off. The carbon black used was a granular material with sizes of about 2 μm to 20 μm, with C element being the main component, as shown in Figure 8e,f. After being rinsed, the carbon black was removed completely both macroscopically and microscopically (Figure 8g,h). The C element was not detected in the main elements, indicating the entire superhydrophobic surface had been completely cleaned (Figure 8i). The results showed that the superhydrophobic superalloy surface had an excellent self-cleaning property for carbon black.

#### 3.5.2. Self-Cleaning of High-Temperature Carbon Deposition

**Figure 9:**
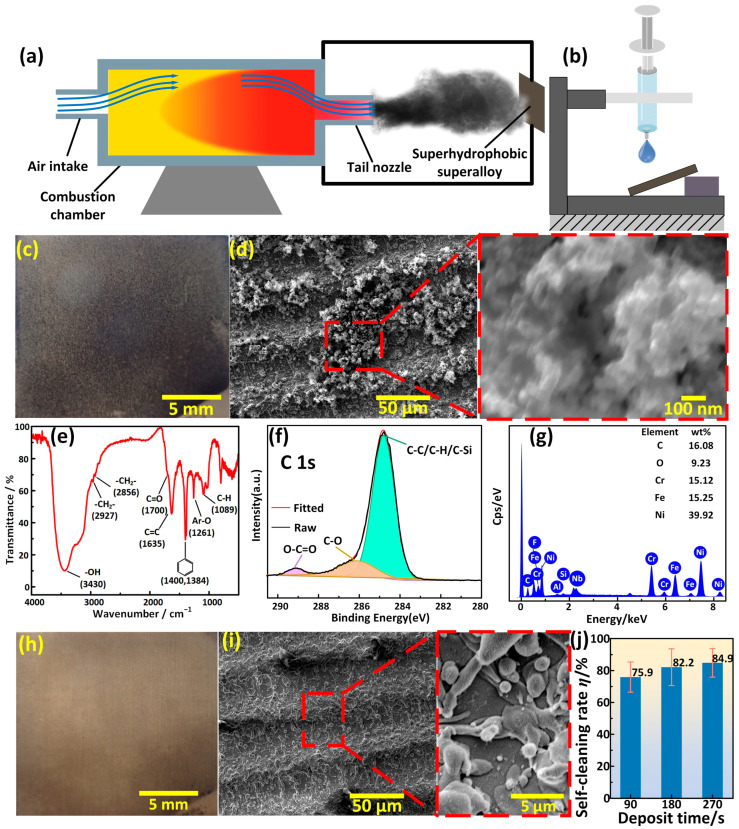
Self-cleaning of high-temperature carbon deposition. (**a**) Schematic illustration of the experimental system for the high-temperature carbon deposition. (**b**) Schematic illustration of the self-cleaning system. (**c**,**d**) Macro- and micromorphology of the superhydrophobic superalloy surface upon which carbon was deposited for 180 s. (**e**,**f**) FTIR spectrum and XPS of the carbon deposition. (**g**) EDS of the superhydrophobic superalloy surface after cleaning. (**h**,**i**) Macro- and micromorphology of the superhydrophobic superalloy surface after cleaning. (**j**) Variation of the self-cleaning efficiency with deposition time. The superhydrophobic superalloy surface was processed under the laser processing parameters of 15 W-500 mm/s-50 μm.

In order to verify whether the superhydrophobic superalloy surface has a self-cleaning property against the carbon deposition produced by the combustion of fuel at high temperature, another self-cleaning experiment was carried out. Figure 9a shows the schematic illustration of the experimental system, in which diesel was used as fuel and discharged after combusting at high temperature in the combustion chamber. A large amount of carbon was deposited on the surperhydrophobic surface, and the amount of carbon deposition on the superhydrophobic superalloy surface was controlled by adjusting the deposition time. Due to the carbon particles adhering to the surface, the roughness of the sample surface was further increased, resulting in a slight improvement in hydrophobicity. The CA, RA_‖_, and RA_⊥_ changed to 163.7 ± 0.8°, 3.8 ± 0.9°, and 3.6 ± 1.3°, respectively. After deposition, the surface was tilted 30° and cleaned with 30 μL of water droplets dropped from a height of 20 cm to wash the carbon deposition away, as shown in Figure 9b. Figure 9c shows the macromorphology of the superhydrophobic surface on which carbon was deposited for 180 s, and the entire surface was completely blackened, indicating a considerable carbon deposition on the surface. The micromorphology of the superhydrophobic surface with carbon deposit indicated the carbon deposition had micron-sized cluster structures, as shown in Figure 9d. Enlarged SEM image showed that these micron-sized cluster structures were actually composed of carbon particles with a size of 50 nm. The compositions of the carbon depositions were characterized with FTIR and XPS, respectively, as shown in Figure 9e,f. Some typical vibration peaks of some functional groups of carbon deposition were detected from the FTIR spectrum, such as -CH_2_-, and C=O, C-H. In addition, the peaks of C-C/C-H, C-O and O-C=O group could be observed in the XPS. According to previous research, the results indicated that the carbon deposition was actually a typical product of diesel combustion, composed of organic hydrocarbon matter and inorganic carbon.

Although tiny carbon particles with a size of only 50 μm could easily fill the gaps of the micro/nanostructures on the superhydrophobic surface, follow-up experiments showed the superhydrophobic surface still exhibited a superior self-cleaning performance. Water droplets rolled off the surface and took away the carbon deposition easily, thereby refreshing the surface. Figure 9g is the EDS spectrum of the superhydrophobic superalloy surface after self-cleaning, which shows the contents of various elements on the surface returned to the state before deposition (Figure 8c). The photo shown in Figure 9h indicated that most of the carbon deposition was cleaned. The micro/nanostructures formed by the deposition of molten superalloy were completely exposed again and not covered by carbon deposition, as shown in Figure 9i. After the self-cleaning experiment, the CA, RA_‖_, and RA_⊥_ of the superhydrophobic surface were 162.7 ± 1.2°, 4.8 ± 1.5°, and 5.2 ± 1.6°, respectively, indicating that its hydrophobicity was not destroyed and could be reused multiple times. By comparing the mass of cleaned off carbon deposition with the mass of deposited carbon deposition, the calculation formula of self-cleaning rate could be obtained, as shown in Equation (1):

(1) $$\eta =\frac{m_{2}-m_{3}}{m_{2}-m_{1}}\times 100\%$$

where, *η* is the self-cleaning rate; *m*_1_ is the initial mass of the superhydrophobic superalloy sample, mg; *m*_2_ is the mass of the superhydrophobic superalloy sample after the deposition of carbon, mg; and *m*_3_ is the mass of the superhydrophobic superalloy sample after water rinsing, mg.

Although the amount of carbon deposition under different deposition times was different, at least 75% of the carbon deposition could be cleaned (Figure 9j).

**Figure 10:**
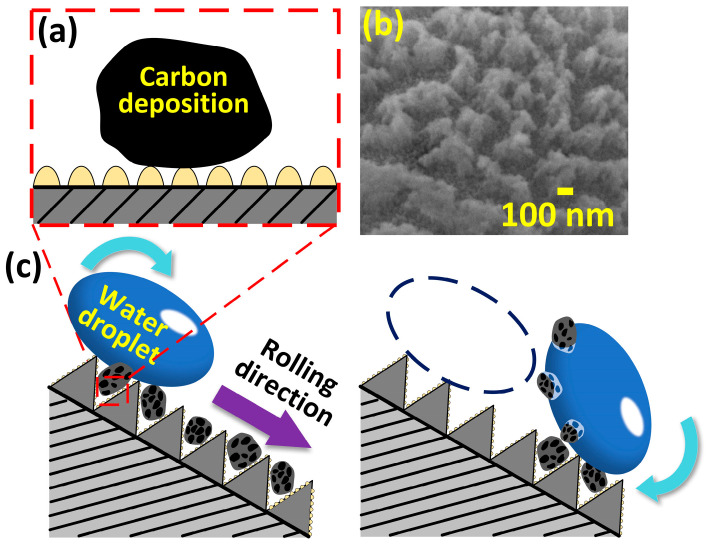
Self-cleaning mechanism of the superhydrophobic superalloy surface. (**a**) Ultrahigh-resolution SEM image of the superhydrophobic superalloy surface. (**b**) Schematic illustration of the contact between the carbon deposition particles and superhydrophobic superalloy substrate. (**c**) Schematic illustration of the self-cleaning mechanism.

The self-cleaning property of carbon deposition on the superhydrophobic superalloy surface was analyzed. The carbon deposition particles had a size of only about 50 nm, which was seemingly easy to deposit and grow in the gaps of the superhydrophobic micro/nanostructures. However, the micro/nanostructures produced by the deposition of molten superalloy were covered by finer structures with a size of only about 20 nm, which reduced the contact area and contact force between the carbon deposition particles and the substrate directly, as shown in Figure 10a,b. Therefore, during the self-cleaning experiment, the carbon deposition particles were easily carried away from the gaps of the micro/nanostructures under the effect of the surface tension of the water droplets, resulting in a self-cleaning effect mimicking the lotus leaf (Figure 10c).

## 4. Conclusions

In summary, we proposed a simple method to fabricate superhydrophobic superalloy surfaces by laser processing and low-surface-energy modification. To ensure the superhydrophobicity and high mechanical strength of the surface, the laser-to-surface energy input should be minimized by optimizing the processing parameters including power, scanning speed, line spacing, and number of scans. The laser power and scanning speed both affect the width and depth of each laser-affected region. Only when the depth exceeds 10 μm can the surface have the possibility of obtaining superhydrophobicity. The line spacing determines the degree of overlap between adjacent laser-affected regions. When the line spacing is greater than the width of the laser-affected region, adjacent processing areas are independent and do not overlap, leaving uncovered areas on the sample surface without micro/nanorough structures. When the line spacing is smaller than the width of the laser-affected region, the sample surface is completely covered by micro/nanorough structures. Scanning the sample more than once will exacerbate the damage of surface and not improve its superhydrophobicity. Under the laser processing parameters of power-15 W, scanning speed-500 mm/s, and line spacing-50 μm, the surface scanned by the laser only once could obtain superhydrophobicity with a 164.5° water CA and a 4.4° water RA. A single laser scan is sufficient to construct the microstructures required for superhydrophobicity while minimizing the loss of mechanical strength. The mechanical strength of the specimens scanned only once was basically the same as that of the specimens without laser scanning and greater than that of the specimens scanned 10 and 200 times. Artificial carbon black and carbon deposited at high temperature were both easily taken away by water droplets, demonstrating the excellent self-cleaning property of the superhydrophobic superalloy surface. The fabrication of a superhydrophobic superalloy surface with high mechanical strength using nanosecond lasers paves the way for the development of metal parts with self-cleaning, anticorrosion, and antifouling properties and provides the possibility for the application of the superhydrophobic surfaces in the fields of aviation, aerospace, and automotive engineering.

## Figures and Tables

**Table 1:** Summary of CAs and the contaminant reported in recent publications on laser-textured metals and polymer substrates.

No.	Substrate	Laser	CA	Contaminant	Reference
1	1A99 Al alloy	Femtosecond laser	152°	Dust (20~40μm)	Su et al. [35]
2	304 SS	Fiber nanosecond laser	152 ± 1.3°	Iron powder (98~106 μm)	Wan et al. [36]
3	304 SS	Femtosecond laser	~150°	Sponge particles	Yao et al. [37]
4	PTFE	CO_2_ laser	168.36°	Dust	Zhan et al. [38]
5	Silicone rubber	Nd:YAG nanosecond laser	159 ± 1°	Dust	Patil et al. [39]
6	SS 304 L	UV laser	~154°	Dust	Wang et al. [40]
7	Al 2024	Nanosecond laser + picosecond laser	161 ± 2°	MnO_2_ (1 μm, 100 μm), PA (~100 μm)	Milles et al. [41]

**Table 2:** Chemical compositions of K424 superalloy (wt.%).

C	Cr	Co	W	Mo	Al	Ti	Fe	Nb	V	Si	Mn	Ni
0.14–0.2	8.5–10.5	12.0–15.0	1.0–1.8	2.7–3.4	5.0–5.7	4.2–4.7	2.0	0.5–1.0	0.5–1.0	0.4	0.4	Bal.

**Table 3:** The CAs and RAs of water droplets under different laser processing parameters.

	1700 mm/s		1300 mm/s		900 mm/s		500 mm/s		100 mm/s	
	*d*/μm	CA/°	*d*/μm	CA/°	*d*/μm	CA/°	*d*/μm	CA/°	*d*/μm	CA/°
	*h*/μm	RA_‖_/°	*h*/μm	RA_‖_/°	*h*/μm	RA_‖_/°	*h*/μm	RA_‖_/°	*h*/μm	RA_‖_/°
	*L*/μm	RA_⊥_/°	*L*/μm	RA_⊥_/°	*L*/μm	RA_⊥_/°	*L*/μm	RA_⊥_/°	*L*/μm	RA_⊥_/°
**30 W**	87.02 ± 2.28	149.3 ± 2.1	91.25 ± 3.20	146.1 ± 3.7	98.08 ± 2.24	158.3 ± 2.3	**105.40 ± 4.44**	**160.2 ± 1.6**	**116.65 ± 3.35**	**151.4 ± 1.9**
	5.72 ± 0.38	None	6.93 ± 0.46	None	8.87 ± 0.91	None	**12.57 ± 1.31**	**5.2 ± 0.8**	**76.06 ± 3.95**	**10.4 ± 0.9**
	85	None	90	None	95	None	**100**	**7.9 ± 0.8**	**115**	**16.9 ± 0.9**
**15 W**	73.37 ± 2.13	125.1 ± 4.6	77.42 ± 1.59	127.0 ± 5.0	85.08 ± 3.27	142.6 ± 2.2	**90.67 ± 3.05**	**164.5 ± 0.9**	**98.80 ± 5.06**	**161.9 ± 2.2**
	3.80 ± 0.15	None	4.68 ± 0.22	None	6.19 ± 0.52	None	**10.32 ± 0.64**	**4.4 ± 0.3**	**42.78 ± 2.04**	**4.8 ± 0.9**
	70	None	75	None	85	None	**90**	**4.4 ± 0.7**	**95**	**5.8 ± 0.7**
**1.5 W**	54.31 ± 2.82	102.0 ± 4.9	54.27 ± 1.77	106.3 ± 2.4	54.30 ± 2.41	108.2 ± 1.5	55.75 ± 1.41	111.0 ± 5.9	**50.38 ± 1.18**	**157.1 ± 1.7**
	3.14 ± 0.17	None	3.87 ± 0.17	None	5.07 ± 0.35	None	5.92 ± 0.40	None	**15.24 ± 1.23**	**15.0 ± 0.8**
	50	None	50	None	50	None	50	None	**50**	**16.6 ± 1.1**

## Data Availability

Data are contained within the article.
